# Detoxification of pulping black liquor with *Pleurotus ostreatus* or recombinant *Pichia pastoris* followed by CuO/TiO_2_/visible photocatalysis

**DOI:** 10.1038/s41598-018-21597-2

**Published:** 2018-02-22

**Authors:** Claudia M. Rivera-Hoyos, Edwin D. Morales-Álvarez, Juanita Abelló-Esparza, Daniel F. Buitrago-Pérez, Nicolás Martínez-Aldana, Juan C. Salcedo-Reyes, Raúl A. Poutou-Piñales, Aura M. Pedroza-Rodríguez

**Affiliations:** 1 Laboratorio de Microbiología Ambiental y de Suelos, Grupo de Biotecnología Ambiental e Industrial (GBAI) Departamento de Microbiología, Facultad de Ciencias, Pontificia Universidad Javeriana, Bogotá, DC Colombia; 2 Laboratorio de Biotecnología Molecular, Grupo de Biotecnología Ambiental e Industrial (GBAI), Departamento de Microbiología, Facultad de Ciencias, Pontificia Universidad Javeriana, Bogotá, DC Colombia; 3 Departamento de Química, Facultad de Ciencias Exactas y Naturales, Universidad de Caldas. Manizales, Caldas, Colombia; 4 Laboratorio de Películas Delgadas y Nanofotónica, Departamento de Física, Facultad de Ciencias, Pontificia Universidad Javeriana, Bogotá, DC Colombia

## Abstract

Cellulose-pulping requires chemicals such as Cl_2_, ClO_2_, H_2_O_2_, and O_2_. The black liquor (BL) generated exhibits a high chemical oxygen demand (COD), five-day biochemical oxygen demand (BOD_5_), and chlorophenol content, along with an augmented colour and increased pH. BL is often discharged into water bodies, where it has a negative impact on the environment. Towards that end, laccases are of great interest for bioremediation, since they can degrade aromatic and non-aromatic compounds while reducing O_2_ to water instead of H_2_O_2_. As such, we evaluated *Pleurotus ostreatus* and *Pichia pastoris* (which produces rPOXA 1B laccase) in the treatment of synthetic BL (SBL) in an “*in vitro*” modified Kraft process followed by CuO/TiO_2_/visible light photocatalysis. Treating SBL with *P. ostreatus* viable biomass (VB) followed by CuO/TiO_2_/visible light photocatalysis resulted in 80.3% COD removal and 70.6% decolourisation. Toxic compounds such as 2-methylphenol, 4-methylphenol, and 2-methoxyphenol were eliminated. Post-treated SBL exhibited low phytotoxicity, as evidenced by a *Lactuca sativa* L seed germination index (GI) > 50%. Likewise, SBL treatment with *P. pastoris* followed by VB/CuO/TiO_2_*/*visible light photocatalysis resulted in 63.7% COD removal and 46% decolourisation. Moreover, this treatment resulted in the elimination of most unwanted compounds, with the exception of 4-chlorophenol. The *Lactuca sativa* L seed GI of the post-treated SBL was 40%, indicating moderate phytotoxicity.

## Introduction

Paper and cardboard production requires various raw materials, such as cane bagasse, wood chips, and sawdust^1,2^. Raw materials are fragmented and treated to obtain cellulose fibres with a low lignin content. This process is performed in two stages; first, wood chips are treated with water, sodium hydroxide, sodium sulphate, and calcium carbonate at a high temperature and pressure, generating Kraft pulp^3,4^. Second, the pulp is separated from black liquor (BL), which is treated as an industrial waste. BL can also be sent to a reagent recovery cycle. In addition, some of the chemical compounds used in the pulping step are recycled^5^. In the second stage, the generated brown pulp then undergoes a bleaching process^3,6,7^.

The effluents produced during the pulping process present high chemical oxygen demands (CODs; 1000–10000 mgL^−1^) and five day biological oxygen demands (BOD_5_ 500–5,000 mgL^−1^). In addition, the effluents result in the dark brown coloration of water bodies, with colour units (CU) between 1,000–8,000. Moreover, due to the use of sodium hydroxide, the pH oscillate between 6.5–11.0. Among the contaminants, the effluents can contain chlorophenols (20–50 mgL^−1^)^2,6^, if these pollutants are discharged either accidentally or intentionally into surface water bodies, they can have a serious impact on the environment. Specifically, polluted wastewater consumes dissolved oxygen, blocks the passage of sunlight, and inhibits photosynthesis. Furthermore, some chlorinated compounds are bioaccumulated and are toxic to various species^8–10^.

To treat contaminated effluents, industries implement primary (physical and chemical) and secondary (microbiological aerobic and anaerobic) treatments to purify wastewater. Such treatments efficiently decrease COD, BOD_5_, sedimented solids (SS), and total suspended solids (TSS)^1,6,11,12^. However, certain chlorinated aromatic compounds and dyes are not completely eliminated. Therefore, industries must evaluate other alternatives to comply with the laws of each country. As such, innovative technologies for recalcitrant material degradation via non-conventional methodologies, such as recombinant microorganisms or fungi that produce enzymes capable of degrading lignin and cellulose, are required^7,9,13^.

Ligninolytic fungi produce various extracellular enzymes (laccase E.C. 1.10.3.2, lignin peroxidases E.C. 1.11.1.14, and manganese peroxidases E.C. 1.11.1.13), which degrade low molecular weight compounds (chelants) and reactive oxygen species (hydroxyl radicals), and are involved in the degradation of lignin (biopulping) and compounds with similar structures such as chlorophenols, modified lignin, and dyes among others^2,7,14–16^. Multiple authors have demonstrated that ligninolytic fungi are a promising alternative for the treatment of wastewater from the paper industry^16–20^.

Recent studies have shown that the direct use of laccase can lead to the rapid and significant degradation of substrates^20,21^. However, laccase production from native sources is limited, since most fungi secrete these enzymes as secondary metabolites under oxygen or other nutrient limiting conditions. Therefore, enzyme production can be slow and difficult to regulate^22,23^. For practical applications, it is necessary to produce laccases not only at low cost, but also more quickly and in higher concentrations than is possible using natural secretions by fungi^24^.

In order to solve these limitations, the use of recombinant microorganisms is a valid option if suitable promoters and secretion signals are used to direct enzyme production in the culture medium^25,26^. Previous studies have evaluated the expression of recombinant laccase from filamentous fungi and yeast in Kraft pulp delignification or cellulose pulp bleaching^27–29^. However, the use of recombinant laccase for the treatment of BL obtained in the pulping process has not been common.

Emerging technologies (such as photocatalysis) for residual water treatment are compatible with other physical and chemical processes, allowing for improved efficiencies and the removal of recalcitrant compounds and their intermediates. Moreover, the combination of traditional and emerging technologies can diminish the initial concentrations of certain contaminants to facilitate contaminant detoxification more effectively than a single process.

Photocatalysis with TiO_2_ is an advanced oxidation technology that has been studied for residual water treatment^30,31^. Although it is highly efficient, it requires the use of ultraviolet light to promote electron excitation, which is monetarily and energetically costly. To mitigate this disadvantage, materials that modify the energy of the TiO_2_ banned band, have been evaluated; reducing the bandgap to less than 2.8 eV and allowing the use of artificial light (fluorescent lamps) or solar radiation. Some approaches using metals, such as copper and alkaline earth oxides (CuO/TiO_2_, CeO/TiO_2_), exhibit high responses in the elimination of toxic organic compounds^32–35^.

We propose a combined treatment using native or recombinant microorganisms followed by CuO/TiO_2_/visible photocatalysis as a sequential process, to promote the *in situ* production of ligninolytic enzymes capable of degrading various aromatic compounds with different degree of chemical substitutions. In this sequential process, the efficiency of toxic compounds adsorption by microbial biomass could increase. Additionally, costs would be reduced by employing fluorescent light or sunlight instead of ultraviolet light and by employing enzymes (laccase, lignin peroxidases, and manganese peroxidases) instead of chemicals. Furthermore, this process will also eliminate residual chlorinated aromatic compounds and colour. Finally, the number of operating cycles that can be performed before the utility of the viable biomass (VB) expires is also worth evaluating. We performed an *L. sativa* L toxicity test as a proof of concept to demonstrate that the treated BL could be reused as irrigation water. The novelty of this work is related to the combination of recombinant microorganisms with CuO/TiO_2_/visible photocatalysis for the sequential treatment of SBL. Other studies have used microorganisms (native or recombinant) or pure or concentrated enzymes for cellulose pulp bleaching^27,28^. However, to the best of our knowledge, this combination has not been previously evaluated for residual BL treatment.

As such, in this study, we evaluated the use of *P. ostreatus* (P.O) and a recombinant strain of *P. pastoris* (P.P), X33/pGAPZαA-*LaccPost-Stop*, producer of the heterologous laccase POXA 1B from *P. ostreatus*^25^, for the treatment of wastewater produced during the alkaline pulping of sawdust residues from *Pinus caribaea*. Furthermore, the number of operation cycles that the two fungi could support was determined, as well as the effect of post-treated effluents on the *L. sativa* L seed germination index (GI). Additionally, complementary removal tests were performed in association with CuO/TiO_2_ fluorescent light photocatalysis to evaluate the toxic compound elimination and the reduction in adverse effects on *L. sativa* seed germination.

## Results and Discussion

### Characterisation of synthetic black liquor

Synthetic black liquor (SBL) exhibited high colour units (CU), chemical oxygen demand (COD), total organic carbon (TOC), and lignin concentrations; lignin was solubilised using sodium hydroxide, high pressure, and temperature during the pulp separation. SBL was characterised by a dark brown colour, possibly due to the presence of chromophores difficult to degrade, such as phenolic aromatic rings, conjugated with carbonyl groups (C=O), quinones, and free radicals among other groups^1^. High absorbances at λ_254_ nm, λ_280_ nm_,_ and λ_465_ nm were also observed, which could be related to aromatic compounds, lignin, and other chromophores. Additionally, SBL exhibited high COD, TSS, SS, and TDS^11,36^ (Table 1). The concentration varied when SBL was diluted. Additionally, to promote mycelial growth and improve enzyme activity, the culture medium was adjusted to a concentration of 5.0 gL^−1^ glucose to meet the nutritional requirements of each microorganism.

**Table 1:** Characterization of synthetic black liquor (SBL).

Parameter	100% (v/v)	10% (v/v)*	10% (v/v)**	5% (v/v)**	1% (v/v)**
pH	7.0 ± 0.2	7.0 ± 0.2	6.5 ± 0.2	6.5 ± 0.3	6.5 ± 0.1
TSS mgL^−1^	3352 ± 12	807 ± 21	934 ± 42	541 ± 33	213 ± 65
SS mgL^−1^	45 ± 3.2	14 ± 1.8	13 ± 1.9	6.4 ± 0.7	2.1 ± 0.3
COD mgL^−1^	50000 ± 234	7250 ± 866	51166 ± 125	29200 ± 230	13666 ± 126
TOC mgL^−1^	7730 ± 256	873 ± 22	ND	1846 ± 89	1543 ± 51
Lignin mgL^−1^	2789 ± 567	350 ± 65	1166 ± 288	1066 ± 115	400 ± 10
CU λ_465 nm_	99521 ± 879	12058 ± 760	29671 ± 345	15656 ± 437	6439 ± 178
Absorbance at λ_465 nm_	1.345 ± 0.34	0.678 ± 0.02	0.789 ± 0.01	0.126 ± 0.02	0.067 ± 0.02
Absorbance at λ_280 nm_	3.876 ± 0.30	1.765 ± 0.56	1.987 ± 0.45	1.175 ± 0.34	0.872 ± 0.03
Absorbance at λ_254 nm_	2.45 ± 0.87	1.98 ± 0.065	1.23 ± 0.031	0.935 ± 0.1	0.567 ± 0.02

Elevated concentrations of CU, COD, TOC and lignin were obtained, mainly from lignin solubilisation in 100% SBL, as a product of the alkaline extraction process. Additionally, aliphatic compounds of different molecular weights were detected. These compounds could affect the COD, TSS, and SS. Elevated absorbency values at different wavelengths could be associated with the presence of aromatic compounds, lignins, and chromophore groups.

Asterisks in the table indicate the following: *Concentration of SBL supplemented for *P. ostreatus*. **Concentration of SBL supplemented for *P. pastoris*. TSS (total suspended solids), SS (sedimented solids), COD (chemical oxygen demand), TOC (total organic carbon), CU (colour units). All data are expressed as averages ± SD.

### Effect of SBL concentration on the removal capacity of *P. ostreatus* and *P. pastoris*

**Figure 1:**
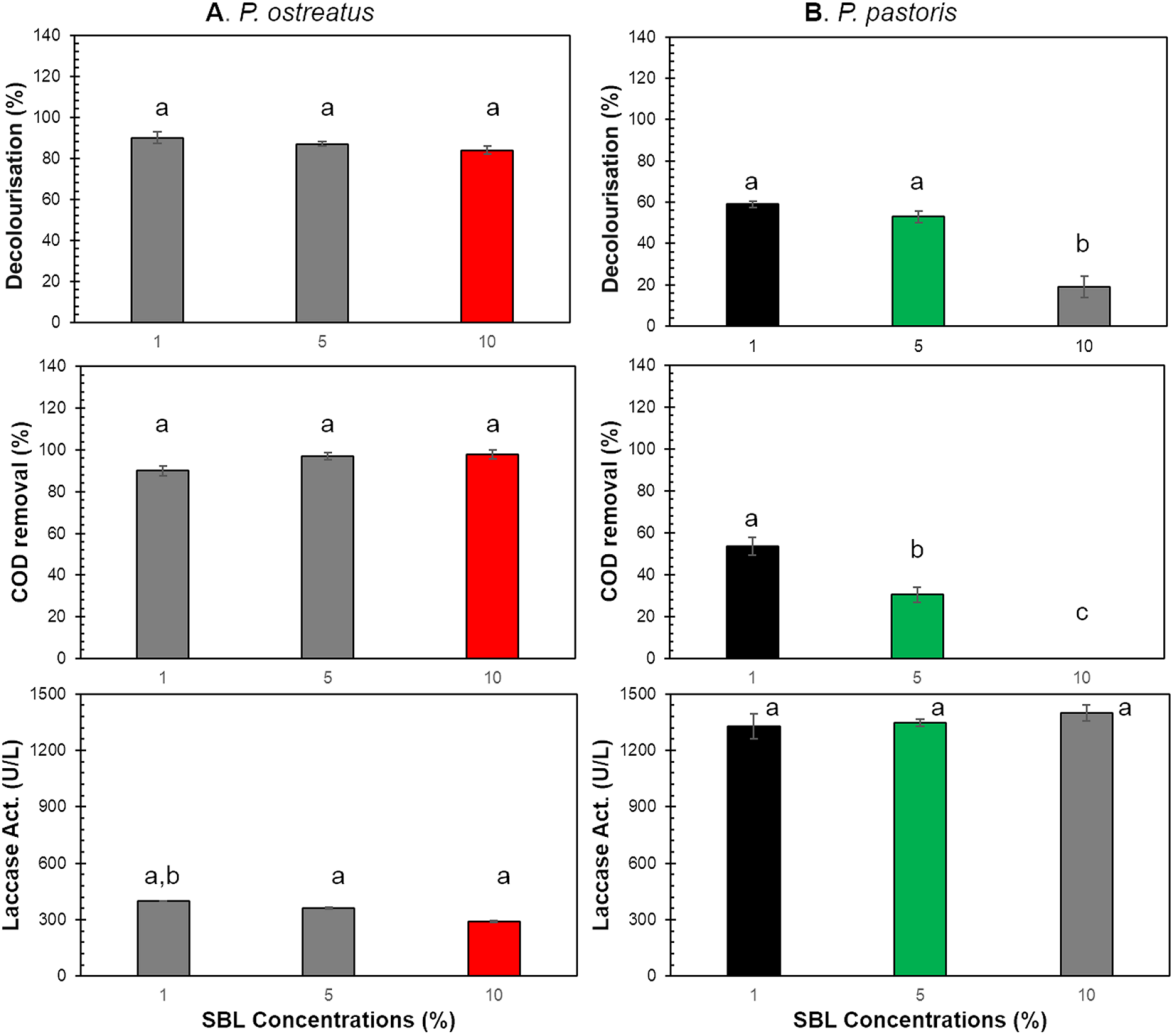
Effect of synthetic black liquor concentration on the removal capacity of *P. ostreatus* and *P. pastoris*. SBL percentages of 1, 5, and 10% (v/v) were evaluated. (**A**). Experiment with *P. ostreatus*. Decolourisation percentages of 90, 87, and 84% (v/v) were observed for 1, 5, and 10% (v/v) SBL, respectively. COD removal values of 90, 97, and 98% were determined for 1, 5, and 10% (v/v) SBL, respectively. Laccase activity ranged between 290 UL^−1^ to 400 UL^−1^ for all evaluated concentrations. (**B**) Experiment with *P. pastoris*. Decolourisation percentages of 59, 53, and 19% (v/v) were observed for 1, 5, and 10% (v/v) SBL, respectively. COD removal values of 53, 30, and 5% were determined for 1, 5, and 10% (v/v) SBL, respectively. The laccase activities for the three concentrations evaluated were 1,327, 1,346, and 1,399 UL^−1^. The letters a,b, and c represent Tukey homogeneous subsets. The letter a corresponds to the best result, followed in order by b and c. Mean ± SD (n = 3).

We found that initial SBL concentration plays a key role in the removal capacity of both native (*P. ostreatus*) and recombinant strains (*P. pastoris*). Preliminary tests with *P. ostreatus* demonstrated that the native fungus efficiently removed more than 50% of the colour and COD (data not shown) of SBL at different concentrations (100, 75, 50, and 25% (v/v)). However, with 10, 5, and 1% (v/v) SBL, higher removal (98, 97, and 90% COD removal) was observed with decolourisation percentages of 84, 87, and 90%, respectively. The laccase activity ranged from 290 to 400 UL^−1^ at the three evaluated SBL concentrations (Fig. 1A).

*P. pastoris* rPOXA 1B was more sensitive to high SBL concentrations (100, 75, 50, and 25% (v/v)), and no removal was observed (data not shown). Thus, we challenged the recombinant microorganism with lower SBL concentrations of 10, 5, and 1% (v/v). After 5 days, the decolourisation was 19, 53, and 59%, respectively. Regarding COD elimination, the removal percentages were 5, 30, and 53%, at 10, 5, and 1% SBL, respectively. The laccase activities at 10, 5, and 1% (v/v) SBL were 1,399, 1,346, and 1,327 UL^−1^, respectively (Fig. 1B).

When comparing the bioremediation by *P. ostreatus* at different SBL concentrations, significant differences were observed in decolourisation, COD removal, and laccase activity (*p* > 0.0001), demonstrating that the native strain could be used to remove organic matter at all evaluated concentrations. Therefore, only the highest SBL concentration (10% v/v), similar to that of Kraft pulping liquor obtained in a mill, was used for the subsequent removal curve assays^1,37^. Similarly, with *P. pastoris*, significant differences in the decolourisation and COD removal (*p* < 0.0001) were observed with different concentrations of SBL. 10% SBL (v/v) resulted in the lowest removal percentage (<50%). Therefore, the removal curve assays were only performed with 5 and 1% (v/v) SBL.

The removal capacity of *P. ostreatus* is associated with the native fungus ligninolytic enzyme system. This process involves different laccases, H_2_O_2_-dependent enzymes (manganese peroxidase, and lignin peroxidase), and hydrogen peroxide-generating systems^24,38,39^. The ligninolytic system can act on BL, white liquor, or lignosulfonates, which are rich in phenolic compounds with various molecular weights, via successive oxidation reactions to form cationic radicals, aliphatic compounds, and possible mineralization leading to CO_2_ production^40^. Additionally, *P. ostreatus* biomass can partially remove SBL by adsorption to the fungal wall, exhibiting an additional physicochemical mechanism not associated with primary metabolism and augmenting the removal efficiency^41,42^.

The genome of filamentous fungi, in particular Basidiomycetes, contains multiple genes encoding laccases. The expression of these genes can be induced by the addition of analogous substrates, copper ions, or lignin-related aromatic compounds. Moreover, adding nutritional factors, such as carbon and nitrogen sources, can induce laccase expression^43^. However, the presence of an inducer does not necessarily guarantee the expression of all encoded genes. The genome of *P. ostreatus* itself encodes 12 laccase genes. However, it has been determined that in liquid cultures, only two laccase genes are expressed in high proportions, namely LCC1 and LCC2^44^.

In contrast, recombinant *P. pastoris* only produces laccase rPOXA 1B^25^, which partially acts on phenolic aromatic compounds; other ligninolytic enzymes are required to obtain greater SBL contaminant removal. However, the contact time between SBL and *P. pastoris* (rPOXA 1B producer) at evaluated concentrations can influence the removal efficiency. The adsorption capacity of *P. pastoris* (producer of rPOXA 1B) was lower than that of *P. ostreatus*, most likely as a consequence of the differences in yeast vs. fungus size and morphology. In addition, differences in the cell wall affect removal based on adsorption phenomenon, as they can result in diminished potential active sites.

### Decolourisation, chemical oxygen demand, total organic carbon (TOC), and lignin removal of SBL effluent by *P. ostreatus* and *P. pastoris*

**Figure 2:**
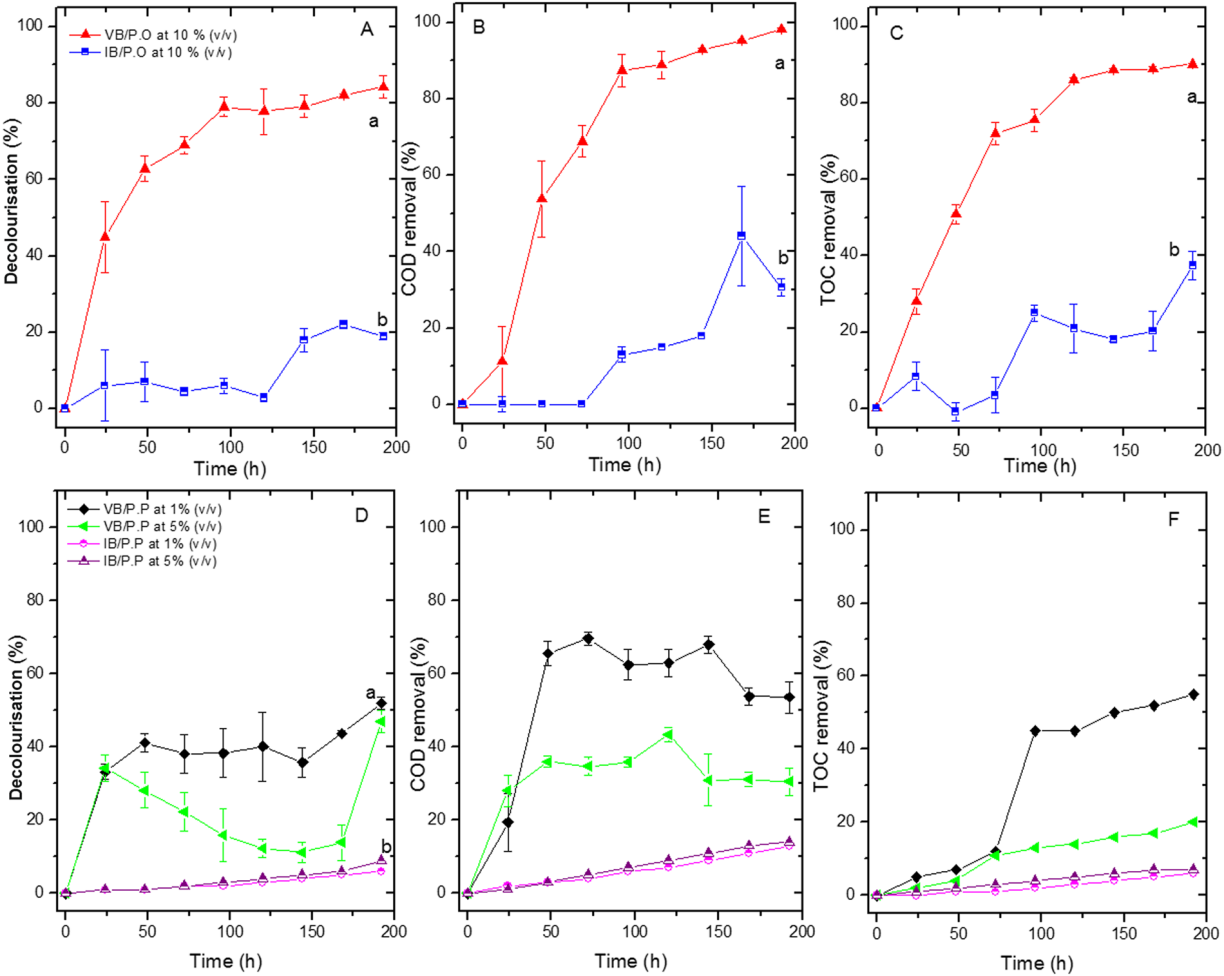
*P. ostreatus* viable biomass (VB/P.O) and inactive biomass (IB/P.O) removal curves. (**A**) Decolourisation. (**B**) COD removal. (**C**) TOC removal. *P. pastoris* viable biomass (VB/P.P) and inactive biomass (IB/P.P) removal curves. (**D**) Decolourisation. (**E**) COD removal. (**F**) TOC removal. For all cases, after 192 h of treatment, significant differences were observed between VB/P.O and IB/P.O, (*p* < 0.0001), with decolourisation values of 84 and 19% and COD removal of 90 and 37%, respectively. For *P. pastoris*, VB removal percentages for 5 and 1% SBL increased gradually with time, with higher values for VB than for IB. Decolourisation values of 47.5 and 52% were observed, along with COD removal of 30 and 53% and TOC removal of 20 and 55%. Lower values were observed for IB: 9 and 6.3% for decolourisation, 14 and 13% for COD removal and less than 10% for TOC. The letters represent heterogeneous groups obtained after Tukey test analysis. The letter a corresponds to the best treatment, followed by the letter b. The bars represent the Mean ± SD (n = 3) for consistency.

Figure 2A–C present the removal percentages for the three dependent variables obtained with *P. ostreatus* after 192 h of treatment. Significant differences were observed between *P. ostreatus* viable biomass (VB) (VB/P.O) and inactive biomass (IB) (IB/P.O), (*p* < 0.0001), with maximum percentages of 84% (VB/P.O) and 19% (IB/P.O). Similar significant differences were observed for COD and TOC removal (*p* < 0.0001), with values of 98% and 90% respectively at 192 h for VB/P.O, which were higher than the values obtained with IB/P.O (30 and 37% for COD and TOC, respectively). On the other hand, significant positive correlations were observed between decolourisation and removal of COD and TOC (R^2^ = 0.934, 0.977, and 0.989, respectively) with a value of *p* < 0.05 for the three parameters.

The *P. pastoris* viable biomass (VB/PP) and inactive biomass (IB/PP) with 5 and 1% (v/v) SBL is presented in Fig. 2D–F. For *P. pastoris*, the decolourisation percentages increased as a function of time, until decolourisation values of 47.5 and 52% were achieved with 5 and 1% SBL, respectively. The maximum COD removal was 53% for VB/PP with 1% SBL, and a removal value of 30% was observed for VB/PP with 5% SBL. Finally, the TOC removal with VB/PP with 1% SBL reached 55%, whereas for VB/PP, a maximum removal of 20% was attained with 5% SBL. These values were significantly higher than those obtained with IB/PP with 5 and 1% SBL (9 and 6.3% for decolourization and 14 and 13% for COD, respectively) (*p* < 0.0001). TOC removal did not exceed 10% with 5% and 1% SBL. A significant positive correlation was observed between the three variables with 1% SBL (R^2^ = 0.81, 0.80, and 0.81 for decolourization, COD, and TOC removal, respectively) with *p* values of 0.047, 0.049, and 0.042. With 5% SBL, a positive, but insignificant correlation was observed (*p* > 0.05).

**Figure 3:**
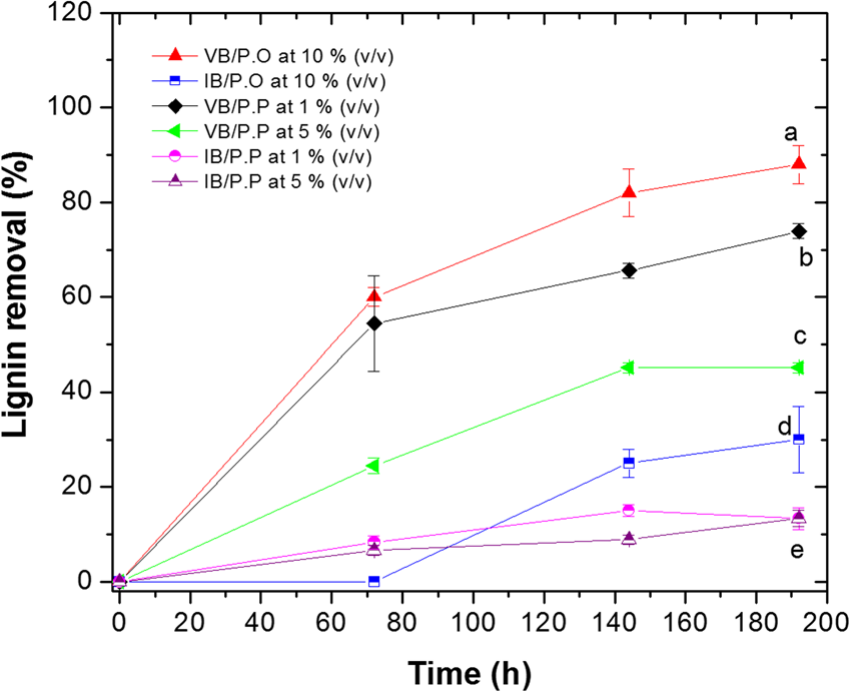
Lignin removal for *P. ostreatus* viable biomass (VB/P.O), inactive biomass (IB/P.O), *P. pastoris* VB/P.P and IB/P.P with 10%, 5%, and 1% (v/v) SBL. Lignin removal was observed for both microorganisms, reaching levels of 88% for 10% (v/v) SBL with *P. ostreatus* and 73% for 1% (v/v) SBL with *P. pastoris*. The removal by adsorption for both cases ranged between 13 and 30%. The letters represent heterogeneous groups obtained after analysis with the Tukey test. The letter a, corresponds to the best treatment, followed by the letters b,c,d and e. The results are presented as the mean ± SD (n = 3).

Lignin removal (%) was analysed at 72, 144, and 192 h. A high removal was observed with VB of the two fungi, reaching percentages of 88 and 73% for *P. ostreatus* and *P. pastoris* with 10 and 1% (v/v) SBL, respectively. The removal for the two microorganisms ranged from 13 to 30% (Fig. 3) for IB/P.O and IB/P.P.

*P. ostreatus* can modify lignin polymers. Dashtban *et al*. (2010) reported that white rot fungi can simultaneously perform enzymatic and non-enzymatic degradation of this polymer, producing simultaneous decreases in colour, COD, and TOC in wastewater from the paper industry. Polyphenol oxidases, haemoperoxidases, and accessory enzymes that generate hydrogen peroxide, such as glyoxal oxidase (E.C. 1.2.3.15), aryl alcohol dehydrogenase (E.C. 1.1.1.90), and quinone reductase (E.C. 1.6.5.5) participate in these enzymatic degradations. The combined action of these enzymes allows for partial or total delignification, forming free radicals, which can continue to oxidise the products to give low molecular weight intermediates. In non-enzymatic degradation, during the delignification process, the fungi lower the pH by releasing hydrogen peroxide, which can react with iron salts to generate hydroxyl radicals with high oxidizing power (the biological Fenton reaction). This reaction indirectly favours the oxidation of aromatic rings to produce carboxylic acids and CO_2_. Furthermore, other sources of C and N were added to the SBL as nutritional supplements. The fungus can assimilate these nutritional sources for biomass formation and energy production, resulting in decreased COD, TOC, and TSS^40^.

Sharma *et al*. (2014) reported additional strategies involving fungi with cellulase and hemicellulase production that were useful for effluent bioremediation in the paper industry. Although the enzymes were not quantified in the present study, Rojas-Higuera *et al*. (2017) reported that when grown in pine, oak, and eucalyptus sawdust, this fungus produces endoglucanases (E.C. 3.2.1.4), endoxylanases (E.C. 3.2.1.8), β-glucosidases (E.C. 3.2.1.21), and cellobiohydrolases (E.C. 3.2.1.91). The production of these enzymes could increase COD and TOC removal^45^. Another factor that may favour COD and TOC removal is a high C/N ratio in SBL compounds. In this study, SBL was supplemented with carbon (5.0 gL^−1^ glucose), favouring fungal metabolism. Similar results were reported by Pedroza-Rodríguez *et al*. (2013), who found that by gradually increasing the paper effluent C/N ratio during treatment with immobilised *Trametes versicolor* biomass, COD removal (>80%) and decolourisation (>80%) were favoured^46^.

When comparing the results obtained in this work with those of other studies, differences between white rot fungi of the same genus may be noted. Freitas *et al*. (2009) evaluated the effect of *Pleurotus sajor-caju* on COD, lignin, and colour removal in paper effluents. After 10 days of treatment, removal percentages of 72, 79, and 72% were observed. These values were lower than those obtained in this study; moreover, two additional days of treatment were required.

In contrast, the removal capacity of *P. pastoris* is associated with the production of a single laccase (rPOXA 1B), as well as the use of glucose and yeast extract (nutritional supplementation of SBL) as carbon and nitrogen sources for primary metabolism. Under these conditions, the laccase enzyme was able to generate changes in aromatic rings with -OCH_3_ groups. In addition, changes or stretches in the C=O bonds of conjugated carbonyls and acid carboxyl groups were also observed^47^. These modifications could account for the lower decolourisation, decreased COD removal, and diminished TOC removal. Although they did not reach the values achieved by *P. ostreatus*, they are important considering that only a ligninolytic enzyme (rPOXA 1B) was present.

### Laccase activity, glucose consumption, and pH

**Figure 4:**
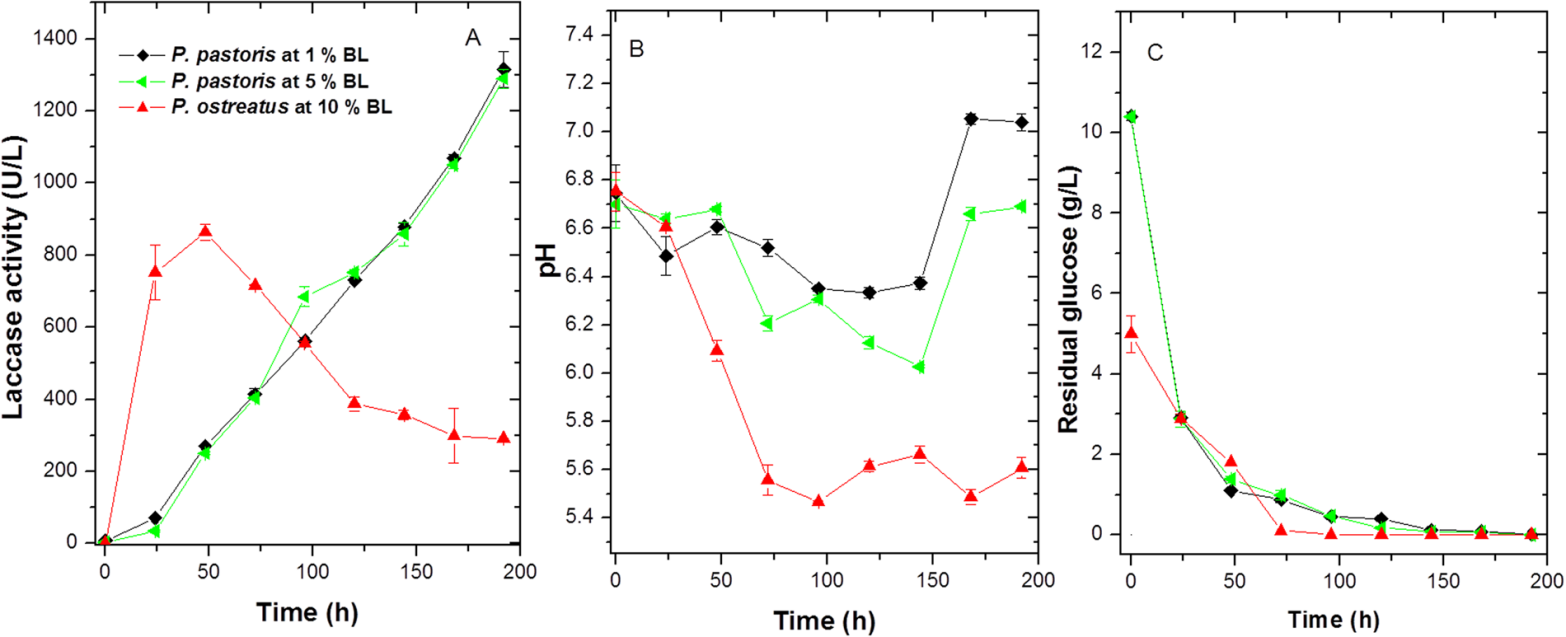
(**A**) Laccase activity, (**B**) pH, and (**C**) residual glucose for *P. ostreatus* in 10% (v/v) SBL and *P. pastoris* in 1% and 5% (v/v) SBL. The laccase activity depended on the microorganism. The removal curves for *P. ostreatus* showed an increase in activity during the first 48 h, reaching a maximum value of 862 UL^−1^, followed by a decrease at 192 h with an activity of 290 UL^−1^. In contrast, the constitutive expression regulated by the laccase p*GAP* promoter enzyme was constant in the *P. pastoris* removal curves for both SBL concentrations, with activities of 1,346 and 1,327 UL^−1^ for 5 and 1% SBL, respectively. Regarding glucose consumption and pH variability, both fungi gradually consumed the carbon source, generating a decrease in pH attributed to organic acid production. The results are presented as the mean ± SD (n = 3).

Different laccase activities were observed between yeast and fungus. For the *P. ostreatus* removal curves, an increase was observed up to 48 h of processing, where a value of 862 UL^−1^ was obtained. Subsequently, at 192 h, the activity decreased to 290 UL^−1^. This could suggest that phenolic compounds present in the SBL induced the activity of laccases in *P. ostreatus*. The enzymes could oxidise and demethylate aromatic compounds with subunits containing methoxyl groups at carbons 3 and 5. Other enzymes, such as peroxidases, could have generated aliphatic intermediates and carboxylic acids downstream^47^. Therefore, no positive (significant) correlations between laccase activity and colour removal, COD, TOC, and lignin (p = 0.051, 0.057, 0.067, and 0.071, respectively) were observed with *p* > 0.05 (Fig. 4A).

Native laccase induction by aromatic compounds alters the enzyme activity as a function of time and relationship with peroxidases. Using *T. versicolour*, Pedroza-Rodríguez and Rodríguez-Vázquez (2013) showed that when the fungus was in contact with the effluent from paper pulping industries, laccase and manganese peroxidase activity could be quantified. Both enzymes were related to colour removal, COD, and dechlorination of chlorinated aromatic compounds^46^.

Compared to *P. ostreatus*, the heterologous laccase production by *P. pastoris* as a function of time was different. Enzyme activity increased up to 192 h, regardless of SBL concentration, with final activities of 1,346 and 1,327 UL^−1^ with 1 and 5% SBL, respectively. This result was associated with the constitutive expression system used to clone the enzyme^25^. In *P. ostreatus*, laccase production varied over time and was influenced by the presence of different inducers. *P. pastoris* rPOXA 1B production is regulated by the *P*_GAP_ promoter, which controls the expression of glyceraldehyde 3-phosphate dehydrogenase (GAPDH, E.C. 1.2.1.12). GAPDH is involved in one of the most important steps of the glycolytic pathway, and is constitutively expressed in the presence of several carbon sources, such as glucose, glycerol, oleic acid, and methanol^48^. Therefore, the constitutive expression of rPOXA 1B laccase in SBL media supplemented with glucose (5.0 gL^−1^ glucose) accounted for the increase in laccase activity throughout the treatment.

At all time points, the laccase activities of recombinant *P. pastoris* were higher than those obtained with *P. ostreatus*. Additionally, the laccase activity showed a significant positive correlation with all variables at both SBL concentrations (R^2^ values above 0.9 and *p* values below 0.05). As confirmation, the only laccase expressed in *P. pastoris* was involved in the removal of some compounds present in SBL (Fig. 4A).

In relation to glucose consumption and pH variation, both microorganisms gradually consumed the carbon source, resulting in the production of organic acids, thus decreasing the pH. However, an important aspect of *P. pastoris* rPOXA 1B laccase is the recombinant enzyme stability at different pH values and temperatures. Previously, this enzyme was reported to be stable at pH 4.0 for 1 h (>90% relative activity)^49^. Furthermore, at pH values between 2.0 and 9.0, the recombinant laccase maintained relative enzymatic activity >60% for 1 h. In addition, it was stable for 1 h at temperatures between 10 and 70 °C, with a relative enzymatic activity of >50%. These results confirmed the possibility of using rPOXA 1B laccase with different residues for numerous applications, due to its activity at different pH values and temperatures^49^.

Glucose consumption by *P. ostreatus* could favour the production of organic acids and hydrogen peroxide, which are necessary for manganese and lignin peroxidase activity (Fig. 4B,C). The production of the *P. pastoris* recombinant laccase POXA 1B is directly regulated by *P*_GAP_, which is associated with glucose metabolism and biomass production (behaviour is depicted in Fig. 4A,C)^25^. Therefore, residual glucose in the medium decreased as a result of yeast metabolism, with a concomitant gradual increase in recombinant laccase production over time.

### Gas Chromatography Mass Spectrometry (GC-MS)

GC-MS analyses of initial SBL and SBL post-treated with *P. ostreatus* or *P. pastoris* VB are presented in Table 2. The compounds identified in untreated SBL were 2,4-methylphenol RT = 10.47, 2,4-dichlorophenol RT = 11.36, and 2,6-dimethoxyphenol RT = 15.96. The presence of some chlorophenols compounds could be related to the extraction process. Under these conditions, free aromatic rings are chemically unstable and may undergo chlorination by small quantities of free chlorine present in water. Both microorganisms transformed chlorinated and non-chlorinated phenolic compounds. However, the compound peak shape differed between the native and recombinant strains.

**Table 2:** Phenolic compounds identified by GC-MS in initial SBL and in samples post-treated with *P. ostreatus* and *P. pastoris*.

RT (min)	Detection of compounds					Compounds
	a	b	c	d	e	
7.83	—	—	+	—	+	2-chlorophenol
8.90	—	+	+	—	—	2-methylphenol
9.29	—	+	+	—	—	4-methylphenol
10.28	—	—	—	—	—	2-nitrophenol
10.47	+	—	—	—	—	2,4-dimethylphenol
11.36	+	—	+	—	—	2,4-dichlorophenol
13.26	—	—	—	—	—	2,3,5-trichlorophenol
13.59	—	—	—	—	—	2,4,5-trichlorophenol
14.68	—	+	+	—	—	2-methoxyphenol (guaiacol)
15.96	+	—	—	—	—	2,6-dimethoxyphenol (siryngol)
16.38	—	—	+	—	—	2-methoxy-4-ethyl-phenol (4-ethyl guaiacol)
19.04	—	—	—	—	—	3-aryl-6-methoxyphenol (m-eugenol)

The presence of chlorophenol could be related to the alkaline extraction process to obtain SBL. Under this condition, free aromatic rings were unstable and could be chlorinated by the presence of small quantities of Cl_2_ in the water. Both fungi were able to biotransform chlorinated and non-chlorinated phenolic compounds, although the modifications by the native fungus and recombinant yeast were distinct.

RT: Retention time (min); **a**: Initial phenol in SBL; **b**: SBL treated with *P. ostreatus* for 192 h; **c**: SBL treated with *P. pastoris* for 192 h; **d***:* SBL treated with *P. ostreatus* and Cu/TiO_2_ for 10 h; **e**: SBL treated with *P. pastoris* and CuTiO_2_ for 10 h; ^+^present; ^−^absent.

Comparative analysis of untreated SBL vs. SBL post-treated with *P. ostreatus* showed the absence of the following compounds in post-treated SBL: 2,4-dimethylphenol, 2,4-dichlorophenol, and 2,6-dimethoxyphenol. Enzymes produced by *P. ostreatus* may have demethylated 2,4-dimethylphenol at positions 2 and 4, generating 2-methylphenol and 4-methylphenol.

The dechlorination of 2,4-dichlorophenol at positions 2 and 4 was also demonstrated. The demethoxylation at position 6 of dimethoxyphenol to form 2-methoxyphenol, which was not present in the initial SBL, was also revealed. This compound is considered one of the final aromatic intermediates in lignin degradation and has reported bactericidal and inhibitory effects on many seeds and plants^45^ (Table 2).

A variety of chlorinated and non-chlorinated phenolic compounds were obtained in SBL post-treated with *P. pastoris*, including 2-chlorophenol, 2,4 dichlorophenol, 2-methylphenol, 4-methylphenol, 2-methoxyphenol, and 2-methoxy-4-ethylphenol. The presence of 2-methylphenol, 4-methylphenol, and 2-methoxyphenol in both post-treated SBL samples suggested the biotransformation of precursors 2,4-dimethylphenol and 2,4-dimethoxyphenol by laccase. These findings can be supported by the fact that only one laccase (rPOXA 1B) was expressed by the yeast *P. pastoris*. This laccase likely cannot perform other specific modifications to lignin or phenolic compounds present in SBL. In contrast, the possible presence of several laccases in *P. ostreatus* and other ligninolytic enzymes could be related to the differences in the phenolic compounds in the samples, as determined by GC-MS.

The dechlorination capacity of recombinant *P. pastoris* was different from that of *P. ostreatus* in regard to chlorinated aromatic compounds, since 2,4-dichlorophenol and 2-chlorophenol were detected in *P. pastoris*. This result suggests the following: i) rPOXA 1B laccase might lack a dechlorination cycle; ii) the recombinant laccase requires additional processing time; or iii) this enzyme cannot perform dechlorination at position 4.

The literature reveals that ligninolytic enzymes can perform different processes such as oxidation, demethylation, reduction of benzoquinones, hydroxylation, and reductive dehalogenation of chlorinated aromatic compounds^50^. Moreover, the number and position of chlorine atoms (ortho-, meta-, and para-) can limit the biotransformation capacity and reaction rate^51^.

### Determination of GI for *Lactuca sativa* L seeds

*L. sativa* is among the 10 plants approved by the United States Environmental Protection Agency (EPA)^52^, the International Organization for Standardization (ISO)^53^, and the Organization for Economic Cooperation and Development (OECD)^54^ for the determination of ecological effects of toxic substances. Assays employing GI for *Lactuca sativa* L seeds are simple, fast, reliable, and inexpensive, and they do not require sophisticated equipment. In addition, these assays are based on plants, which are more sensitive to environmental stress than other organisms^55,56^. This germination assay demonstrates the response of a vegetal biological model in the presence of initial SBL compounds and the final SBL compounds after treatment with *P. ostreatus* or *P. pastoris* VB. The GI index serves as a reference to evaluate effluent toxicity in a vegetal organism. Values below 50% indicate that the effluent has high phytotoxicity to the vegetal model^57^.

**Figure 5:**
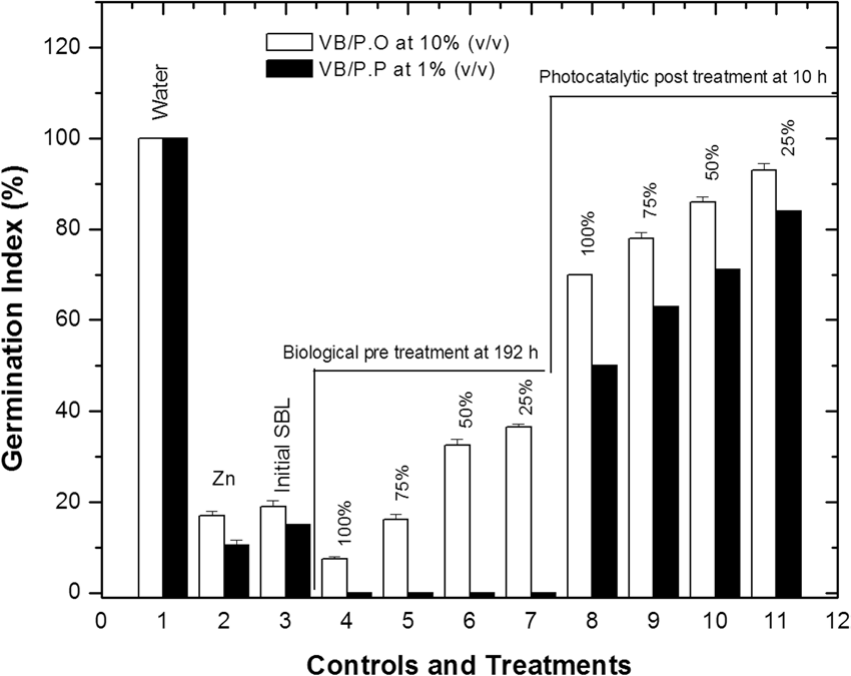
Germination index for *P. ostreatus* and *P. pastoris* viable biomass at 192 h (section A of the graph). Untreated SBL exhibited high phytotoxicity (GI: 18.96%). A similar response was observed for the positive control with ZnSO_4_ (16.9%). After 192 h of *P. ostreatus* VB treatment, GI decreased to 7.47%, indicating higher toxicity than untreated SBL. As the SBL was diluted, the GI percentages increased without exceeding 50%. In the *P. pastoris* experiments, the phytotoxic effect was greater than that obtained for *P. ostreatus*. For this fungus, the GI values were zero for all assayed SBL concentrations (100, 75, 50, and 25% v/v). Germination indexes for *P. ostreatus* and *P. pastoris* effluents after treatment with visible-light photocatalysis using Cu/TiO_2_ for 5 h (section B of the graph). Experiments with SBL post-treated with *P. ostreatus*/CuO/TiO_2_ photocatalysis with visible light gave GI >50%, indicating that the final phytotoxic effect was reduced. Experiments with *P. pastoris*/CuO/TiO_2_ under visible photocatalysis attained a GI of 40%, indicating a moderate phytotoxic effect. All bars represent the mean ± SD (n = 3).

The untreated SBL had a high phytotoxicity (GI of 18.96%), which was similar to that obtained for the ZnSO_4_ positive control (16.9%). The index decreased to 7.47% after treatment with *P. ostreatus* VB for 192 h, indicating a greater phytotoxicity than at the beginning of the experiment. As the effluent was diluted, germination rates increased, but did not exceed 50% (Fig. 5). Therefore, SBL post-treated with *P. ostreatus* VB continued to exhibit an important phytotoxic effect.

Furthermore, SBL samples post-treated with *P. ostreatus* contained compounds such as 2-methylphenol, 4-methylphenol, and 2-methoxyphenol, as detected by GC-MS. These low molecular weight compounds can cross seed cell walls, affecting some biochemical processes related to germination^8^ (Table 2).

The germination assays showed that the phytotoxic effect after SBL treatment with *P. pastoris* was even higher than after treatment with *P. ostreatus*. The germination index was zero at all concentrations (100, 75, 50, and 25% v/v), possibly resulting from intermediates, as revealed by GS-MS. As shown in Table 2, the following compounds were detected in SBL treated with *P. pastoris*: 2-chlorophenol, 2,4-chlorophenol, 2-methylphenol, 4-methylphenol, 2-methoxyphenol, and 2-methoxy-4-ethylphenol. The combination of these compounds could have potentiated the adverse effects on the seeds. On the other hand, it has also been demonstrated that at 50 and 100 mgL^−1^, 2,4-chlorophenol exhibits a phytotoxic effect on *L. sativa* L seeds^58^.

### Operating cycles

The results obtained with *P. ostreatus* demonstrated that VB could be reused for three continuous cycles of operation, equivalent to 576 h, given that no significant differences were observed between cycles for decolourisation, COD, TOC, and lignin removal (*p* values < 0.0001). The laccase activity and radial growth of *P. ostreatus* revealed that the fungus was functional for three continuous cycles of operation, favouring removal processes involving biotransformation and adsorption. The decreased enzyme activity and reduced biomass growth in cycles 4 and 5 could be associated with biomass saturation and the formation of intermediates, which were adsorbed on the fungal wall and resulted in an inhibitory effect on growth and enzymatic activity^42,59^ (Table 3).

**Table 3:** Operating cycles: percentage of decolourisation with *P. ostreatus and P. pastoris* VB.

Parameter	C1	C2	C3	C4	C5
Viable *Pleurotus ostreatus* at 10% (v/v) SBL					
COD (%)	94 ± 0.4^a^	94 ± 0.3^a^	94 ± 0.3^a^	69 ± 1^b^	66 ± 2^b^
Lignin (%)	78 ± 1.2^a^	82 ± 2^a^	70 ± 1.5^a^	51 ± 0.5^b^	37 ± 3.8^c^
Colour removal (%)	78.3 ± 0.8^a^	77.5 ± 1.1^a^	74.7 ± 0.7^a^	68.2 ± 2^b^	31.5 ± 1^c^
Growth (mm)	90 ± 9^a^	80 ± 5^b^	66 ± 5^c^	20 ± 0.1^e^	10 ± 0.3^f^
Laccase act. (UL^−1^)	1159 ± 51^a^	1028 ± 29^b^	1018 ± 34^c^	628 ± 16^d^	596 ± 41^d^
Viable *Pichia pastoris* at 1% (v/v) SBL					
COD (%)	87.1 ± 5.5^a^	87 ± 3.5^a^	84.3 ± 5.0^ab^	79.7 ± 7.0^b^	53 ± 1.8^c^
Lignin (%)	76 ± 2.8^a^	68 ± 1.6^ab^	61 ± 1.2^b^	46 ± 5.7^c^	30 ± 2.6
Colour removal (%)	61 ± 1.7^a^	58 ± 1.9^ab^	18 ± 1.2^c^	17 ± 0.9	5.3 ± 0.5
Growth (Log_10_ CFUmL^−1^)	6.7 ± 0.01^a^	6.7 ± 0.4^a^	6.5 ± 0.6^a^	6.9 ± 0.4^a^	5.5 ± 0.2^b^
Laccase act. (UL^−1^)	1315 ± 49^a^	1126 ± 11^a^	1091 ± 9^b^	803 ± 19^c^	602 ± 49^d^

Five complete treatment cycles were carried out to estimate the number of cycles for which the biomass from both fungi could be used. The decolourisation percentage, TOC and COD removal, laccase activity, and pH were assessed. *P. ostreatus* viability was determined by PDA radial growth, and *P. pastoris* viability was determined by YPG agar microdrop counts.

C1 (cycle number 1), C2 (cycle number 2), C3 (cycle number 3), C4 (cycle number 4), C5 (cycle number 5), CFU (colony forming units).

*P. pastoris* was used for cycle trials with 1% (v/v) SBL. According to the results, recombinant yeast could be used for COD removal for three consecutive cycles, as no significant differences were observed between the three cycles (*p* > 0.0001). However, colour and lignin removal decreased as the number of cycles increased, which could be related to the accumulation and adsorption of chlorinated compounds (2,4-chlorophenol and 4-chlorophenol). In relation to yeast viability and enzymatic activity, the process could be maintained for four cycles due to the stability of rPOXA 1B laccase^49^. Despite the progressive decrease in yeast viability, the activity of the laccase secreted into the culture medium remained constant.

### Preliminary CuO/TiO_2_ photocatalysis tests

The results obtained in this work with either organism suggest that although the removal of toxic compounds was high, the final treated SBL retained intermediates that could affect other organisms. Therefore, preliminary photocatalysis tests were performed with a CuO/TiO_2_-based composite.

Once it was determined that the composite exhibited a spectral response in the visible range, removal tests were performed. For these tests, SBL treated with *P. ostreatus* VB at 192 h had initial values of COD: 350 mgL^−1^, CU: 1,000, and pH: 5.2 ± 0.2. Furthermore, SBL treated with *P. pastoris* VB at 192 h: had initial values of COD: 4,856 mgL^−1^, CU: 2,125, and pH: 7.5 ± 0.2.

When performing the photocatalytic removal tests for 5 h, 80.3% COD removal and 70.6% decolourisation were obtained for *P. ostreatus*-treated SBL. In the experiments with *P. pastoris*, the COD removal was lower at 63.7%; moreover, a decolourisation of 46% was obtained. Furthermore, COD and CU removal under dark conditions by adsorption to the composite did not exceed 15% for either treatment (Table 4).

**Table 4:** Removal percentages for SBL post-treated with sequential treatments.

Parameter	VB/P.O/CuO/TiO_2_ Visible light	VB/P.O/CuO/TiO_2_ Visible light	VB/P.O/CuO/TiO_2_ in dark/adsorption	VB/P.O/CuO/TiO_2_ in dark/adsorption
Decolourisation (%)	80.3 ± 1.8	63.7 ± 0.56	13.7 ± 0.9	12.3 ± 1.1
COD Removal (%)	70.6 ± 2.9	46 ± 6.0	11.2 ± 0.6	8.4 ± 0.4
Final pH	6.3 ± 0.7	8.4 ± 1.1	5.2 ± 1.1	7.3 ± 0.7

After performing the photocatalytic treatment for 5 h on SBL previously treated with *P. ostreatus*, the COD removal was 80.3%, and the decolourisation percentage was 70.6%. For *P. pastoris*, photocatalytic treatment resulted in 63.7% COD removal and 40% decolourisation. The removal in the dark by composite adsorption (negative control) did not exceed 15% for either fungi.

COD (chemical oxygen demand), VB (viable biomass), P.O (*Pleurotus ostreatus*), P.P (*Pichia pastoris*).

The differences between the results were related to concentration, CU, and initial pH values, which were lower for *P. ostreatus* than for *P. pastoris*. Under these conditions, the photocatalysis was more efficient with low loads of organic matter. This result is consistent with those reported by Ghaly *et al*.^60^. In their work, they reported that elevated colour and COD prevented radiation passage, resulted in a decreased photonic efficiency, and saturated the semiconductor surface, resulting in deactivation. On the other hand, the pH determines the surface charge of the composite. Thus, at acidic pH, the composite acquires a positive charge and favours the removal of contaminants with a negative surface charge. In contrast, at alkaline pH, the composite is negatively charged, and the removal of contaminants with the opposite charge is favoured. Both phenomena likely occurred in this experiment, since the SBL had a different pH (Table 1). However, in SBL treated with *P. pastoris*, the efficiency was also affected by the initial contaminant concentration. The oxidation of chlorinated and non-chlorinated aromatic compounds could be carried out by hydroxyl radicals and other strongly oxidizing species. However, detailed studies are needed to understand the production dynamics and mechanisms involved in the oxidation.

To verify whether the photocatalytic treatment produced any changes in the compounds generated by the two organisms, sample analysis (GC-MS) was performed after 5 h of photocatalysis. Under visible-light photocatalysis, all components detected in post-treated SBL with *P. ostreatus* (2-methylphenol, 4-methylphenol, and 2-methoxyphenol) were eliminated. A similar result was obtained for *P. pastoris*, where five of the six initial compounds were not detected. The only compound present was 4-chlorophenol with a retention time of 7.83 min (Table 2).

Previous reports have demonstrated that conventional treatments are not effective for the degradation of toxic contaminants and the organic complexes that constitute BL. For example, one recent development in this field is the advanced oxidation process (AOP). TiO_2_ is a catalyst widely used treats organic pollutants in industrial wastewater^61^. Some authors have evaluated photocatalysis for the treatment of pulping BL (100%) by employing C-Dots/TiO_2_^62^ as a visible-light photocatalyst for 3 h. Using this methodology, most organic compounds were removed, except for heterocyclic compounds containing nitrogen atoms. A COD removal percentage of 81.2% was obtained, which was superior to the visible-light photocatalytic effect of pure TiO_2_ and coal-based C-Dots. It is important to highlight that Bo *et al*. (2017) did not refer to CU or CU removal. However, decolourisation was important, since they worked with 100% BL. In contrast, in this study the photocatalysis treatment followed microorganism detoxification. In addition, the SBL concentration was adjusted to attain microorganism tolerance, resulting in 70.6 and 46.0% decolourisation by *P. ostreatus* and *P. pastoris*, respectively. Doping was carried out with CuO, and visible photocatalysis was employed for COD removal, resulting in removal percentages of 80.3 and 63.7% for *P. ostreatus* and *P. pastoris*, respectively.

The GI results also supported the effects of the visible photocatalysis. The SBL post-treated with *P. ostreatus*/CuO/TiO_2_/visible photocatalysis exhibited a germination percentage >50%, indicating a low final phytotoxic effect. The germination index increased as SBL was diluted to the extent that it was comparable to the distilled water control. In contrast, *P. pastoris*/CuO/TiO_2_/visible photocatalysis resulted in a GI of 40% (moderate phytotoxic effect) (Fig. 5). All results indicated that complementary treatment with CuO/TiO_2_/visible photocatalysis improved the final effluent quality and verified this combined approach as a promising alternative that must be further studied. Future studies will optimise the operating conditions and possible scale-up processes will be evaluated.

Even though this combined approach resulted in bioremediation, the limitations include the gradual wear and inactivation of titanium films, as well as the difficulty in scaling up the film production. Since microorganisms are being used instead of their isolated enzymes, depending on the COD, TOC, BOD, and CU concentrations, substantial effluent dilutions may be necessary to reduce toxicity towards the microorganism and avoid system saturation. Reusing treated water is an alternative; however, it is not an attractive solution for engineers. In addition, in this work, only one gene (*POXA 1B*) was cloned into *P. pastoris* yeast, and the laccase expression was less effective than that of *P. ostreatus*, which produced several enzymes, including laccases, manganese peroxidase, and lignin peroxidase.

## Conclusions

Sequential SBL treatment using native or recombinant microorganisms diminished the initial toxicity load in terms of CU and COD, allowing further treatment with CuO/TiO_2_/visible photocatalysis. High CU and COD concentrations are the main limitations of photocatalysis when visible light lamps are utilised, since they lack sufficient energy to generate semiconductor photoexcitation (TiO_2_). Therefore, the semiconductor was modified with CuO to allow treatment with CuO/TiO_2_/visible photocatalysis, improving the visible light results.

It was also established that VB can be reused for several cycles, which is promising since biological systems must maintain their biotransformation potential for multiple cycles to reduce operational costs. Finally, the results obtained on the laboratory scale with both microorganisms demonstrated pulping liquor bioremediation potential for several operational cycles. However, it is necessary to complement the treatment with other technologies to completely eliminate intermediates affecting *L. sativa* L seed germination.

Even though only one plant model was employed (*L. sativa* L), the results suggest that the water could be evaluated for irrigation purposes and should therefore be assessed with other plant species. A desirable treatment should guarantee removal parameters associated with irrigation processes and the effect of post-treated effluents on sensitive species.

The results obtained in this study raise the possibility of using this type of sequential technology in other stages of the paper industry to comply with environmental regulations in each country. Examples include biological pulping coupled to chemical pulping. Moreover, high added value by products can be employed in the chemical and pharmaceutical industries. Additionally, BL can be used as a raw material. All of these possibilities should be explored and could help corporations to strengthen cleaner production programs, while maintaining sustainable and environmentally friendly development.

## Materials and Methods

### Microorganisms and culture media

*P. ostreatus* (P.O), (Pontificia Universidad Javeriana, Colombia) was reactivated at 30 °C for eight days on wheat bran extract-agar (WBEA) with the following composition: 10 gL^−1^ glucose, 5 gL^−1^ peptone, 2 gL^−1^ yeast extract, 0.1 gL^−1^ KH_2_PO_4_, 0.05 gL^−1^ MgSO_4_.7H_2_O, 0.076 gL^−1^ MnSO_4_.H_2_O, 15 gL^−1^ agar, and 175 gL^−1^ wheat bran extract (WBE)^42^.

Biomass production for studies in liquid medium was carried out in WBE broth. The fungus was cultivated for eight days at 30 °C and 120 rpm. Subsequently, the biomass was recovered by filtration, washed three times with sterile distilled water, and stored at 4 °C until use.

For *P. pastoris* (X33/pGAPZαA-*LaccPost-Stop*), biomass production vials were collected from the Master Cell Bank (MCB)^63^ clone 1. After thawing, they were inoculated in a screw-cap glass tube containing 5 mL of sterile YPG-Z medium (1% (w/v) yeast extract, 2% (w/v) peptone, and 2% (w/v) glucose, supplemented with 40 μg mL^−1^ of Zeocin). Tubes were incubated overnight (O/N) at 30 °C and 180 rpm. Then a 500 mL Erlenmeyer flask was inoculated with 100 mL (EWV, effective working volume) of fresh YPG-Z medium and cultured under the same conditions for 12 h. To rule out the presence of contaminating morphologies, the resulting culture was verified by Gram stain^64^ and used as a 10% (v/v) inoculum for the small-scale production of the crude extract (concentrated supernatant) containing recombinant POXA 1B laccase (rPOXA 1B)^65^.

The production of rPOXA 1B on the laboratory scale was performed under pre-optimised culture conditions: 500 mL Erlenmeyer containing 300 mL culture medium (3/5), 10% (v/v) inoculum, 0.1 mM CuSO_4_, 10 gL^−1^ glucose, 20 mM NH_4_SO_4_, 20 gL^−1^ peptone, 15 gL^−1^ yeast extract, for 168 h at 30 °C and 180 rpm^49^.

### Analytical techniques

The chemical oxygen demand^66^ was determined using a closed reflux method employing the commercial HACH Kit with a detection range of 15 − 15,000 mgL^−1^. The TOC (https://www.hach.com/asset-get.download.jsa?id = 7639983811) concentration was determined using the sodium persulfate oxidation technique (HACH Kit 0-20 mgL^−1^), and the colour units (CU) and percentage of decolourisation were estimated using a previously reported methodology^67^. The laccase activity was quantified following the methodology reported by Tinoco *et al*. (2001)^68^. The reducing sugars were quantified using the 3,5-dinitrosalicylic acid technique reported by Miller *et al*. (1959)^69^.

### Black liquor collection and characterization after chemical pulping

Synthetic black liquor (SBL) was prepared by performing an “*in vitro*” modified Kraft process by adding 50 g of *Pinus caribea* sawdust to 500 mL of 5% (w/v) NaOH. Extraction was performed at 15 psi and 121 °C for 15 min. Black liquor was separated from the cellulose pulp by centrifugation at 7,000 × *g* for 20 min. The liquor pH was adjusted with 1.0 M H_2_SO_4_, and the liquor was refrigerated at 4 °C until use. Table 1 shows the initial characterization at 100, 75, 50, 25, 10, 5, and 1% (v/v) SBL.

### Effect of BL concentration on *Pleurotus ostreatus* and *Pichia pastoris* (X33/pGAPZαA-*LaccPost-Stop*) removal capacity

Synthetic black liquor at 100% (v/v) was diluted with distilled water to obtain concentrations of 75, 50, 25, 10, 5 and 1% (v/v). Experiments with *P. ostreatus* were carried out in triplicate in 100 mL Erlenmeyer flasks containing 50 mL of SBL supplemented with 5 gL^−1^ glucose, 0.05 gL^−1^ (NH_4_)_2_SO_4_, and 1 mM CuSO_4_ with a final pH of 7.0 ± 0.2. An inoculum of 5% (w/v) of pelletized biomass was added to each Erlenmeyer flask. The Erlenmeyer flasks were incubated for 192 h at 120 rpm and 30 °C. At the end of the incubation period, the post-treated SBL was recovered by centrifugation at 7,000 × *g* for 20 min. The percentages of decolourisation, COD removal, and laccase activity (UL^−1^) were determined as response variables. The SBL concentration used in subsequent experiments was selected based on the concentrations with removal >50% and maximum laccase activity (UL^−1^).

For the evaluation of *P. pastoris*, SBL was prepared at concentrations of 10, 5, and 1% (v/v), since the preliminary tests determined that the yeast was inhibited at SLB concentrations of 25, 50, 75, and 100% (v/v). Synthetic black liquor was supplemented with 10 gL^−1^ glucose, 20 gL^−1^ peptone, 15 gL^−1^ yeast extract, 0.1 mM CuSO_4_, and 20 mM ammonium sulfate at a final pH of 7.0 ± 0.2. Experiments were performed in triplicate in 500 mL Erlenmeyer flasks containing 300 mL of SBL supplemented and inoculated with 10% (v/v) *P. pastoris* culture. The Erlenmeyer flasks were incubated for 192 h at 120 rpm and 30 °C. At the end of the incubation period, the post-treated SBL samples were recovered by centrifugation at 7,000 × *g* for 20 min. Subsequently, the same response variables were quantified as for *P. ostreatus*.

In addition, to verify yeast viability, decimal dilutions and microdrop cultures were performed on YPG agar, following a previously reported methodology^70^. The SBL concentration to be used in the removal curves was selected in the same manner as for *P. ostreatus*.

### Decolourisation, chemical oxygen demand, and total organic carbon removal of synthetic black liquor effluent by *Pleurotus ostreatus* and *Pichia pastoris*

Removal curves were performed in triplicate using 100 mL Erlenmeyer flasks containing 50 mL of SBL at 10% (v/v) for *P. ostreatus* and 500 mL Erlenmeyer flasks containing 300 mL of SBL at 5% and 1% (v/v) for *P. pastoris*. The synthetic black liquor was supplemented according to the nutritional needs of each microorganism. The inoculum VB percentages and operating conditions were the same as those used in the tolerance tests. For these experiments, periodic sampling was carried out for 192 h, and IB was used as an adsorption control (obtained by sterilization in an autoclave for 15 min at 121 °C and 1 psi)^59^, labelled as inactive biomass of *P. ostreatus* (IB/P.O) and inactive biomass of *P. pastoris* (IB/P.P). The evaluated response variables were the percentage of decolourisation, COD removal, TOC, residual glucose (gL^−1^)^69^, residual lignin (gL^−1^), pH, and laccase activity (UL^−1^)^68^. Additionally, for experiments with VB, GC-MS was performed at the beginning and at the end of the curves.

### Preparation and characterization of CuO/TiO_2_ composite

Following the protocol previously described by Puentes-Cárdenas *et al*. (2016)^71^, CuO/TiO_2_ composite samples were prepared as follows: a 10 gL^−1^ TiO_2_ suspension in water and a 1.25 gL^−1^ CuSO_4_.5H_2_O solution in ethanol were separately sonicated for 1 h each. Subsequently, samples were mixed and sonicated for 15 min. The pH of the resulting suspension was adjusted to 9.0 with 1.0 M NaOH. Afterwards, the solid was separated by centrifugation, washed with distilled deionized water, oven-dried at 40 °C for 12 h, and calcined in air at 450 °C for 1 h. The resulting CuO/TiO_2_ composite was then characterised in terms of specific surface area, pore volume, and average pore diameter using nitrogen adsorption-desorption at 77.35 K, using a gas sorption analyser (Quantachrome, NOVA 4200e series, USA). The specific surface area was calculated from isotherms by the Brunauer-Emmett-Teller (BET) equation, and the pore size distribution was determined using the Barrett-Joyner-Halenda (BJH) method. The morphological and surface characteristics of the CuO/TiO_2_ composite were observed using a field emission scanning electron microscope (FE-SEM, JEOL, JSM-7401F) operated at 20 kV, equipped with an energy dispersive X-ray spectrometry (EDS) system. The chemical analysis of the CuO/TiO_2_ composite was performed via EDS. The analysis of the CuO/TiO_2_ composite crystalline phase was performed via X-ray diffraction (XRD) using a PANalytical X’Pert PRO MOD diffractometer, operated with Cu K𝛼 radiation (wavelength = 1.54050 Å) at 45 kV and 40 mA. XRD patterns were collected in a 2*θ* range from 10 to 90° with a scan rate of 0.026° per 40 s and a step size of 0.026°. The optical properties of the CuO/TiO_2_ composite were studied using UV-Vis spectroscopy in the wavelength range of 240–800 nm.

### Preliminary tests of visible photocatalysis with CuO/TiO_2_

The final physical and chemical characteristics of SBL treated with viable biomass obtained from both microorganisms was used to evaluate whether further decreases in certain parameters could feasibly be achieved by performing a complementary treatment with visible photocatalysis using a CuO/TiO_2_-based composite. The intermediate compound elimination and the increase in *L. sativa* L seed germination rate (GI) resulting from photocatalysis were also assessed^71^.

Experiments were performed in triplicate using 0.5 g of composite (calcined at 450 °C for 1 hour)^71^, which was deposited by evaporation at 50 °C on the base of a 5.0 cm diameter Petri dish. Subsequently, 10 mL of SBL post-treated with VB of *P. ostreatus* and *P. pastoris* was added. Each treatment was irradiated for 5 h, using two 15 W fluorescent lamps. The lamps were installed 10 cm above the glass base to avoid sample evaporation^71^. At the end of the treatment, post-irradiated SBL was recovered by centrifugation at 10,000 × *g* for 10 min, and the percentages of decolourisation and COD removal, the germination index (GI)^59^ and the GC-MS spectrum fragmentation patterns were determined.

### Germination index determination with *Lactuca sativa* L seeds

The effects of the initial SBL (T_1_) and of SBL post-treated with the two microorganisms on the GI of *L. sativa* L seeds^55,56,72^ (SEMICOL^®^, www.semicol.co) were tested following a previously reported methodology^59,73^. The controls and evaluated treatments were as follows: distilled water (1), 0.001 M ZnSO_4_ (2), initial SBL (3), SBL post-treated with VB/P.O (4), and SBL post-treated with VB/P.P (4)^59,73^. In Fig. 1, numbers 5, 6, and 7 correspond to 75, 50, and 25% (v/v) dilutions of post-treated SBL for *P. ostreatus* and *P. pastoris*. The response variable was the GI (%) calculated using equation 1, as proposed by Zucconi *et al*. (1985)^74^:

1 $${\rm{GI}} \% =\frac{G\ast L}{Gc\ast Lc}\ast 100$$

where GI is the germination index (%), *G* is the average number of germinated seeds per analysed sample, *Gc* is the average number of germinated seeds in the negative control, *L* is the average length of the sample radicle (mm), and *Lc* is the average radicle length (mm) in the negative control. The GI value can vary from 0 to 100%.

### Number of operation cycles

To estimate the number of re-use cycles for *P. ostreatus* and *P. pastoris* VB, tests were performed in Erlenmeyer flasks using 10% (v/v) SBL for *P. ostreatus* and 1% (v/v) SBL for *P. pastoris*. In each experiment, the sample was incubated for 5 days, at 30 °C and 120 rpm for *P. ostreatus* and at 30 °C and 180 rpm for *P. pastoris*. At the end of the first cycle, the post-treated SBL was removed, and a new batch of SBL was added to the Erlenmeyer flasks.

The procedure was repeated until five cycles were completed. In each cycle, the percentage of decolourisation, TOC removal, COD, laccase activity (UL^−1^)^68^, and pH were determined. *P. ostreatus* was monitored by radial growth on potato dextrose-agar (PDA, 4 gL^−1^ potato infusion from 200 g, 20 gL^−1^ dextrose, 15 gL^−1^ agar-agar). The viability of *P. pastoris* was determined using a YPG agar microdrop.

### Statistical analysis

Data distribution and normality tests were performed using the Shapiro-Wilk and Levene tests followed by non-parametric Kruskal-Wallis to define the removal efficiency between VB and IB for both fungi (removal curves) and the number of operational cycles sustained by either VB or IB for both fungi. To identify differences among the sample means, a Tukey test was performed. The treatments were compared with a distilled water control for the *L. sativa* L GI assays. A value of *p* < 0.05 (5%, α = 0.05) was considered to be significant. Additionally, the Pearson coefficient was determined for established significant associations among the different parameters monitored during VB treatments with both *P. ostreatus* and *P. pastoris*. For all analyses, SAS 9.0 for Windows was used.
